# Supplementary material for: Transcriptomic Insights into Mechanisms of Early Seed Maturation in the Garden Pea (Pisum sativum L.)
Source: Cells. 2020 Mar 23;9(3):779. doi: 10.3390/cells9030779 (PMC7140803; doi:10.3390/cells9030779)

Zhewan−1(10) vs Zhon

Global Summary

%DE = 0.89

genes with fdr < 0.2 = 541 (209 + / 332 −)

genes with fdr < 0.1 = 521 (201 + / 320 −)

genes with fdr < 0.05 = 487 (176 + / 311 −)

genes with fdr < 0.01 = 366 (115 + / 251 −)

genes in genesets = 0

<FC> = 0

<t−score> = 1972.92

<p−value> = 0

<fdr> = 0.12

Portrait

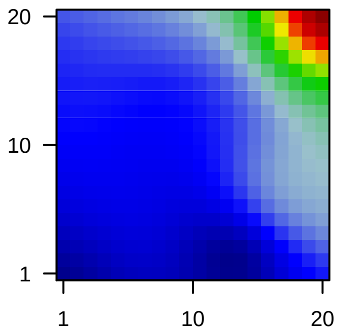

Top 100 DE genes

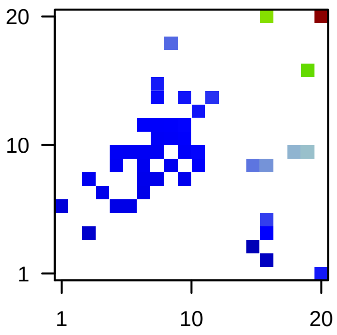

Global Genelist

Rank	ID	log(FC)	p−value / fdr / Metagene	Description
1	TRINITY_DN7	−0.84	0.000000000000000220 x 20	
2	TRINITY_DN6	1.88	0.000000000000000220 x 20	
3	TRINITY_DN1	0.72	0.000000050000000216 x 5	
4	TRINITY_DN4	0.27	0.000000060000000219 x 16	
5	TRINITY_DN1	0	0.00000009000000029 x 11	
6	TRINITY_DN1	0	0.00000010000000027 x 8	
7	TRINITY_DN6	0.75	0.000000130000000416 x 5	
8	TRINITY_DN4	0	0.00000018000000059 x 11	
9	TRINITY_DN1	0	0.00000027000000059 x 12	
10	TRINITY_DN2	0	0.000000390000000510 x 14	
11	TRINITY_DN3	0.26	0.000000470000000519 x 10	
12	TRINITY_DN3	0	0.00000055000000059 x 11	
13	TRINITY_DN3	0	0.00000066000000059 x 11	
14	TRINITY_DN2	0	0.00000066000000059 x 11	
15	TRINITY_DN2	0	0.00000070000000059 x 12	
16	TRINITY_DN3	0	0.00000075000000059 x 11	
17	TRINITY_DN1	0	0.00000083000000053 x 8	
18	TRINITY_DN1	0.81	0.000000890000000516 x 5	
19	TRINITY_DN1	0	0.00000095000000057 x 12	
20	TRINITY_DN1	0	0.00000104000000059 x 11	

p−values

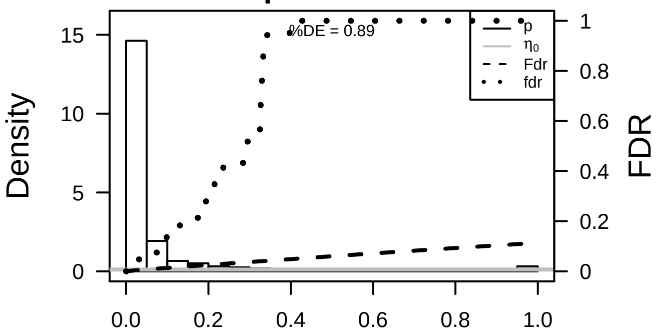

Supplement: Supplementary file 1 [file cells-09-00779-s001.zip › Supplementary materials/FileS3.zip!/FileS2/Hormones - Results/Summary Sheets - Differences/Reports/Zhewan.1.10..vs.Zhongwan.6.10..pdf]
